# *‘They are inconveniencing us’ -* exploring how gaps in patient education and patient centred approaches interfere with TB treatment adherence: perspectives from patients and clinicians in the Free State Province, South Africa

**DOI:** 10.1186/s12889-020-08562-3

**Published:** 2020-04-06

**Authors:** N. Moodley, A. Saimen, N. Zakhura, D. Motau, G. Setswe, S. Charalambous, C. M. Chetty-Makkan

**Affiliations:** 1 The Aurum Institute, Johannesburg, South Africa; 2 College of Medicine and Dentistry, James Cook University, 1 James Cook Drive, Douglas, Townsville, Queensland 4814 Australia; 3 TB Programme, Department of Health, Bloemfontein, Free State Province South Africa; 4 School of Public Health, Faculty of Health Sciences, University of the Witwatersrand, Johannesburg, South Africa

**Keywords:** Tuberculosis TB, Adherence, Messaging, Loss to follow up, Knowledge, Health literacy, Patient-centered care

## Abstract

**Background:**

Tuberculosis (TB) treatment loss to follow up (LTFU) plays an important contributory role to the staggering TB epidemic in South Africa. Reasons for treatment interruption are poorly understood. Treatment interruption appears to be the culmination of poor health literacy of patients and inadequate health education provided by clinicians. We explored clinician and patient perspectives of the gaps in TB messaging that influence TB treatment LTFU.

**Methods:**

We conducted semi-structured in-depth interviews between January and May 2018 with a sample of 15 clinicians managing TB and 7 patients identified as LTFU in public clinics in the Free State Province, South Africa. Thematic analysis using a mixed deductive/inductive thematic approach was used.

**Results:**

Limited occupational opportunities, fear of disclosure and stigmatization all contributed to treatment LTFU. Patients felt that the TB messaging received was inadequate. Many of the clinicians interviewed felt that improving patient’s TB knowledge would reinforce adherence to treatment and thus focused on sharing information on treatment completion, side effects and infection control. However, the inability of clinicians to establish rapport with patients or to identify social support challenged TB treatment adherence by patients. Clinicians perceived this as patients not following their instructions despite what they considered lengthy TB education. Having said this, clinicians concurred that their medical management of TB lacked the psycho-social dimension to treat a social disease of this magnitude.

**Conclusions:**

Limited occupational opportunities, fear of disclosure and stigmatization all contributed to treatment LTFU. Clinicians concurred that poor patient understanding of TB and that biomedical management lacking a psycho-social dimension further exacerbated the poor treatment outcome. TB remains a social disease, the successful management of which hinges on patient-centred care.

## Background

Tuberculosis (TB) remains the leading cause of death from a single infectious agent globally, ranking higher than HIV/AIDS (human immunodeficiency virus/ acquired immunodeficiency syndrome), and despite treatment being available [1]. In 2018, South Africa ranked fourth on the list of high TB burden countries based on absolute number of TB cases with an estimated 520 incident cases per 100,000 population [1]. The fragmented South African health system is largely accountable for the diagnostic delays, inconsistent guideline implementation and poor TB outcomes that mar the TB programme [2]. The comprehensive cascade of care analysis conducted by Naidoo et al *on the 2013 programmatic data revealed that* 5% were unable able to access TB testing, 13% did not receive their TB diagnosis and 12% were never initiated on treatment [3]. However, evidence suggests that these ‘missed’ cases continue to engage with the health system and the system fails to manage them appropriately [3]. In 2016, South African data estimates that 17%of patients initiating TB treatment failed to complete their treatment; an attrition equivalent to 92,761 individuals [3].

TB control efforts are persistently undermined by social, financial and clinical barriers to care [4]. TB treatment adherence is vital in achieving disease cure in individual patients and preventing disease spread within the community [5]. The World Health Organization’s (WHO) has declared the ongoing emergence and spread of drug resistant TB a global crisis, emphasizing the necessity for TB patients to complete their course of TB treatment [1]. Patients incur considerable out of pocket expenses in accessing TB services despite TB diagnosis and care services being offered free in South Africa; with the poorest patients incurring highest relative costs [6]. Poor treatment adherence increases the length and severity of illness and complicates disease transmission and drug resistance; carrying negative economic consequences for the patient, community and the health system [7].

Studies exploring TB treatment completion generally adopt a bio-medical approach, though TB has long been considered a ‘social’ disease [8]. Socioeconomic and individual circumstances including patients’ sense of feeling better, occupational commitments, side effects, and misinformation on treatment duration impact heavily on treatment completion [9–14]. Health education is integrated into services provided at primary health care (PHC) level in South Africa [15]. However, inadequate patient counselling on treatment expectations [16, 17] and inconsistent implementation of the Directly Observed Treatment, Short Course (DOTS) strategy [18, 19] continue to exacerbate TB treatment LTFU. Apart from South Africa, similar findings of poor patient-provider communication hindering TB treatment completion have been demonstrated as far afield as Eritrea, Tanzania, Pakistan and Ethiopia; always with the discussion prioritizing strengthened patient-provider trust and treatment literacy [20–24].

The WHO End TB Strategy has endorsed a holistic patient centred model of care, focused on patient rights and welfare, to improve TB outcomes globally [25–27]. Disseminating accurate, evidence-based TB information among patients and clinicians alike, is fundamental to achieving this. Data from as far back as 1997 purported that TB care in South Africa remained nurse-centred with poor receptivity and confirmation by the patient [28].

Despite the need to empower patients to actively manage their TB, current literature lacks feasible approaches to accomplish this empowerment. This study allows for exploration of the complex social, cultural and environmental phenomena relating to TB treatment LTFU in the Free State Province and identifies potential areas for improvement.

## Methods

### Setting

This study was conducted at three public health facilities in the Mangaung District (Free State Province, South Africa) that served high density, marginalised communities with rife unemployment and poverty [29]. HIV prevalence and TB mortality were estimated at 11 and 9% respectively in the in the district [29]. The incidence of drug sensitive TB among those commenced on TB treatment in the Free State Province (2017) was 429.8 per 100,000, compared with the national average of 378.6 per 100,000 [30]. Tuberculosis was the leading cause of death, irrespective of age or sex, in the Free State Province [31] and HIV-positive TB mortality accounted for 73% of deaths among patients with TB in 2018 [1]. Further, 94% of patients newly diagnosed with TB were initiated on TB treatment and 90% of those patients who were TB/HIV co-infected were able to access anti-retroviral therapy [29]. Patients documented as LTFU regarding their TB treatment and clinicians that managed patients with TB at these facilities were approached to participate in the study.

### Design

This exploratory qualitative study was conducted between January and May 2018 with purposive sampling used to select patients. Three research assistants (RAs) that conducted the in-depth interviews (IDIs) were Good Clinical Practice (GCP) accredited, trained in qualitative data collection, had previous interviewing experience and fluent in the local languages (English, Sesotho, and Setswana). During training, interviewers were assessed and given feedback following direct observations on their ability to collect data using the interview guides. The investigators regularly reviewed the interviews and provided frequent feedback to the research assistants. Debriefing sessions were arranged to discuss experiences of conducting interviews, interview content, how to improve probing skills and address challenges.

The interviewers prior contact with the patients was limited to arranging interviews. The interviewers had engaged with the clinicians previously during quantitative TB work conducted at the facilities. Participants were provided with information on the qualitative component of the study and the interviewer’s role during the interview. The semi-structured interview questions allowed for the successive coding process required for deductive/inductive thematic analysis approach.

### Data collection

Patients and clinicians that were willing to provide written voluntary informed consent and agreed for the session to be digitally recorded were interviewed. Separate interview guides were developed for the patients (Annexure 1) and clinicians (Annexure 2) that explored access to health care, perceived quality of care, TB knowledge and how treatment support impacted TB treatment completion. The interviews were audio-recorded and were between 30 to 90 min. Basic demographic information (e.g. age, level of education, distance to clinic, and source of income, history of co-morbidities) was collected from patients (Annexure 3: Table 1). Information on position of employment and years of experience was collected from clinicians (Annexure 3: Table 2). The interviewers made field notes after the interviews. It was the author’s assessment that saturation was reached by revising the probes during data collection.

Patients listed on electronic TB records from study facilities; aged ≥18 years and documented as LTFU (as per the WHO treatment outcome definition [32]), were invited to participate in the study. The sample varied by age and gender. Patient contact information was retrieved from the clinical records. Initial attempts were made to contact patients telephonically. Failing which, liaison with the local tracing team was undertaken to trace patients using listed addresses. Adherence to standard practise for LTFU tracing was ensured with only the clinical staff of the facility, the contact tracing team and members of the research team who were tasked with locating LTFU patients having access to the patient identity and contact information. Reports submitted contained anonymised data. From a total of 268 patients identified with the TB outcome of LTFU on the ETR.net and EDR.web (electronic TB registers), we were only able to retrieve 71 case files. Sixty-one (61) of these case files contained invalid contact details and we were thus only able to contact 10 patients for interviews. One patient was unable to be interviewed as he had relocated to another province and two had died. The remaining seven patients identified for interviews were approached either face-to-face or telephonically. Five patients chose to be interviewed at their homes and two chose the facility. (Annexure 4). Patients who commuted to the facility for the interview were reimbursed R150 ($10 US) in lieu of travelling expenses. Inadequate collection of patient contact information and treatment records impacts on tracing and management of patients. Sinai et al. demonstrated that it is commonplace for patients to move between facilities once they have commenced their TB treatment [33]. Often these movements occur without prior notice to the provider and rendering patient tracking difficult to trace [3, 34, 35]. Sinai et al. additionally reflected on the data quality issues within the patients’ records, TB screening registers and case registers and with routine laboratory data including incompleteness, errors and discrepancies [33].

Clinical managers at each facility in collaboration with the research team identified clinicians that could be interviewed. Clinicians were currently employed at the selected health facilities; and were part of the nursing staff or doctors that were directly involved in TB care and management. Clinicians were approached face-to-face for participation and interviews were arranged at their convenience. Interviews were conducted in private areas within the facility.

### Data analysis

Recorded interviews were transcribed verbatim and translated to English where applicable by research assistants. Transcripts were checked for accuracy by the research assistants. The transcripts were further checked, and identifiers removed by two investigators with medical and doctorate level qualifications. Manual coding was used for data analysis. To validate the findings, independent coding and theme identification was conducted by the same investigators. A codebook was developed that comprised a priori codes derived from the original question guide. Emerging thematic codes were developed during the thematic analysis and added to the codebook as required. Concepts from different interviews were pooled together and integrated into common themes. For reliability, four additional investigators reviewed the codebook before themes were finalised using the deductive/inductive thematic approach. The themes are represented as direct quotes. The themes were summarised by the authors as they were developed during the analysis and the direct quotes by the participants were used to support the summary.

### Ethics

Ethics approval for this study was obtained through the Human Research Ethics Committee (University of the Witwatersrand) [Ethics approval no: 170606] and the Free State Department of Health [Ethics approval no. FS_2017RP10_005]. Written consent was obtained from all participants. The interview scripts were coded, and personal identifying details were not collected.

## Results

Baseline characteristics of patients interviewed showed a male predominance (86%) with mean age of patients being 37 years (7.8 standard deviations [SD]). Patients travelled on average 9.8 km to the attending facility (Table 1). A total of 15 clinicians with varying experience (two Medical Doctors, one Operations Manager, nine Professional Nurses and three Enrolled Nurses), were recruited for in-depth interviews. The average duration of employment for the clinicians was 17 years (2.7 SD).

### Main theme 1: Patient level perspectives regarding adherence to TB treatment

Most patients reported that they were given TB information by clinicians during their clinical visits. However, it appears that the information received by patients did not translate into improved TB clinical outcomes as evidenced by the inability of this cohort of patients to complete their TB treatment regimen. Often patients demonstrated an understanding of the disease but the social determinants of health at play within the community, such as diminished educational and employment prospects, had far reaching consequences that included TB treatment LTFU, fear of disclosure and stigmatization. Patients felt that the TB messaging received was inadequate. The sub-themes derived from the interviews drew attention to the impact of health literacy on TB treatment outcomes and suggested that the rigid implementation of the current TB treatment guidelines stifled active patient involvement in their ongoing treatment. Commonly reported reasons for LTFU in the literature (including long queues, financial difficulty attending clinic and unreliable transport) were inconsistently reported in this study and were not considered for the analysis.

#### Subtheme 1.1: Gaps in health literacy related to information on TB treatment adherence

Most patients demonstrated adequate knowledge of the TB screening procedures and TB symptoms. However, patients did not seem to understand the TB treatment process and importance of their roles and responsibilities in achieving successful TB outcomes. Most patients described the TB messaging received as focussed on the importance of adhering to TB treatment for the ‘entire duration of therapy’, despite potential side effects of the TB medication. *“Yes, they tell you that you have the TB illness and tell you that if you take your treatment correctly, comply to your treatment you will be fine. They make you feel good that you end up accepting the fact that you really have TB” (#4).* Patients did not seem sufficiently prepared regarding the side effects that were experienced while on TB treatment. *“Yes, they (referring to clinicians) did explain to me but though they have explained something, when it happens to you it is not the same as talking about it, when it is happening, it is you who feels the side effects of the treatment so even when they mention that, they do explain but when you experience it somewhere, that does not sit well.” (#5).*

Importantly, patients seemed to lack knowledge on the length of treatment, nor that the treatment would have to be continued significantly past the point where the patient felt a subjective improvement in health. One patient described his experience as follows: *“She (referring to clinician) did not tell me, she never told me [to stop] but I decided to stop the treatment. She told me that I will be finishing taking treatment on the 12*^*th*^*, they said I should give another sputum to check if TB is still active or what. When I went to the clinic, I produced sputum and I was okay. I was okay, my body weight was back, and it’s when she told me that you will be completing your treatment” (#7).*

#### Subtheme 1.2: Challenge to individual TB treatment plans

Some patient’s decision to discontinue treatment was in response to unmet expectations from clinicians. One patient felt that the rigidity of clinicians in failing to restructure the management plan to accommodate their requests resulted in them interrupting their treatment. *“I was treated well but they could not when I explained to them that I will go away for work for this long (one month) and I am asking for treatment that can last for the time I am away. They could not give me like that, the way I was asking them, that is the only reason that made me to end up defaulting now” (#5).*

Some patients also felt that the clinicians were not listening to their concerns and expected them to adjust to the TB medication. *“I vomited when I took the pills for the first time and I had a bit of diarrhoea. To overcome these, I went back to the clinic and they gave me soft porridge powder and told me that I will get used to the pills and they will get used to my system or body” (#4).*

Patients also felt that some of the existing systems at the public health facility were not conducive to them accessing TB treatment efficiently. *“I am delayed sometimes, I go at five o’clock in the morning and come home at six o’clock in the afternoon. They are inconveniencing us. It is just the fact that when I visited the clinic due to epilepsy, when am I supposed to go that other side [to get TB treatment], they are delaying us, and I would just leave it like that” (#1).* Another perceived obstacle to patients not adhering to their TB treatment plans could be the negative attitude of clinicians towards patients. *“I was not feeling okay and I was coughing while at work and I lost weight and I was taking no TB treatment up until the manager/white guy came to fetch us to come back from there. I went to take my treatment after I came home, and the nurse/sisters reprimanded me there” (#3).*

*“I didn’t like the way she treated me and I did not allow her and looked at me , asked why did I have this and this on my neck, we were quarrelling , both of us you see …*. *I did not do anything, I just left” (#6).*

#### Sub-theme 1.3: Motivators to adhering to TB treatment

It seems that patients could be willing to adhere to their TB treatment if they are given the opportunity to be constructively involved in their health management.

*“Uh, I think what can be done in situations like mine, where it is work related. I think if they can try and do as I pleaded, they should understand our complaints and our reasons because we are asking, and we are willing to get treatment the way we are asking. I think that is the solution that can be done” (#5).*

Patients also seemed to express a need for individual centred counselling which could help them develop and manage their personal TB treatment plan. *“I suffered, and I could not accept the news, but I eventually accepted because it was checked from the lab sir. I eventually accepted because I was counselled. They told me to come for my treatment that is when I believed and accepted that I do have TB” (#4).*

### Main theme 2: Clinician level perspectives regarding patient adherence to TB treatment

Many clinicians felt that improving the patient’s TB knowledge would reinforce adherence to treatment and thus focused on sharing information on treatment completion, side effects and infection control. However, the inability of clinicians to establish rapport with patients or to identify social support challenged TB treatment adherence by patients. Clinicians perceived this as patients not following their instructions despite what they considered lengthy TB education. Having said this, the clinicians concurred that their medical management of TB lacked the socio-economic dimension to treat a social disease of this magnitude.

*“Ehm from the training side with clinicians, ehm mainly its ehm, they emphasise on … as we also know as clinicians that emphasis is mainly on them, informing patients about taking their treatment on a daily basis and the importance and when to take treatment mainly. But training, its, it doesn’t, you know the training that I have attended doesn’t really emphasise on the socio-economic factors of our patient,” (#8).*

The subthemes below highlight how the clinician’s individual interpretation of the national TB guidelines resulted in significantly different key messages being communicated to patients, how the fragmentation of the health system leads to ongoing patient and clinician frustration; and begins to outline the importance of community support in addressing TB.

#### Subtheme 2.1: Lack of standardisation of communication regarding TB therapy

Clinicians felt empowered from the ongoing TB module training aligned to the National TB Guidelines and this was reflected broadly in their understanding of the TB transmission dynamics and their demonstrated competency in TB care provision. However, the ability to transfer this information successfully to all TB patients was possibly lacking. *“I think we still have to work hard, very hard for the patients to understand TB. They really don’t understand it, so there are few that are really understanding very well but I still think we still have a lot of work to do, to teach patients more about TB” (#19).*

Some clinicians anecdotally correlated successful treatment completion with the patient’s knowledge of TB, as they believed this indicated that the patients were taking their illness seriously. *“If they do not know, obviously if you don’t have the reason why you are doing something, obvious it’s not going to do it mos (local South African term indicating an expression) neh but if you have knowledge that you are supposed to take your medication so that you can be better, obviously you will take the medication correctly” (#3).*

During the consultations, few clinicians discussed information on the complications of interrupting or stopping treatment; or regarding the continuation of treatment should patients transfer to other facilities. When transfer letters were negotiated, there was no indication that the clinicians reinforced the dangers of stopping or discontinuing treatment. “*… we do encourage them that when you know that you are going somewhere, come and get your transfer letters so that you can get the continuation of your treatment” (#19).*

#### Subtheme 2.2: Dealing with patient frustration and health system inadequacies

A few clinicians felt that providing TB services at a single focal point allowed experienced staff to effectively relay information on TB transmission, therapy and adherence. This interaction would create the opportunity for patients to express their expectations of clinicians and clinicians would in turn improve patient rapport and establish a supportive, therapeutic relationship. Familiarity and consistency within the health service would reassure the patient and strengthen the trust in the care provided; potentially allowing for the disclosure of difficulties. *“I am like a parent to them, you are free to them, you talk, you educate the person so that he can come back again. When he is having problems though he will not be taking treatment from you, he is able to approach you to ask” (#12).*

Some clinicians felt that the staff rotation hindered rapport with the TB patients due to short rotation periods at the TB focal point. As such, clinicians were unable to gain comprehensive insight into the patients’ perspective and TB messaging could not be personalised to the patients need. Maintaining permanent staff at the TB focal point was described as a reason for reducing treatment interruption at their facility. *“No the thing is, it is impossible, here at the clinic as we are rotating and then the patients come across a lot of faces. There are those who are dependent on that certain sister, you see … he can’t be explaining himself a lot. Is it that when the other one comes in, the patient is supposed to explain himself again and that is taking him backwards” (#12).*

The personal attitudes of staff, often perceived as harsh and condescending by the patients, may have contributed to the failure in establishing rapport with the patient. Often, the inability of the clinician to develop the patient-clinician relationship resulted in abrupt discontinuation of TB treatment without the opportunity of an intervention by the clinician.

*“Unfortunately, people want to be treated by happy people, attitude. So an over worked person sometimes can you know show a bad attitude and that can, maybe he is having good intention, but the patient receives it differently and next time doesn’t want to come back to the clinic, so those are some of the things that can make patients not to come” (#19).*

## Discussion

This exploratory study highlighted the complex interplay of patients’ beliefs, perceptions and health literacy regarding TB treatment adherence and how the perceived ineffective health service delivery colluded to hinder TB treatment completion. A prominent finding of this study were the apparent gaps in communication emanating from the patient and health service. These communication gaps undermine strengthening the patient-centred approach.

Communication experts describe effective communication as a comprehendible, two-way process naming ‘participation’ and ‘dialogue’ as key elements [36]. Patient education is reinforced with communication and social mobilization programmes that together with the broader community, are essential for improving TB cure rates and enhancing sustainability [36]. Most clinicians believed that the TB messaging they delivered was comprehensive and effective and that patients were only partially receptive to the information. There are several dimensions to this statement that should be explored.

Firstly, the clinicians have all described educating their patients. However, there appears to be evidence to the contrary regarding the quality of information given by clinicians to patients. Christian et al. explored the quality of HIV and TB health care provision in the Western Cape and its alignment to the national protocols. They cited that only 43% of cases met the minimum threshold for adequate case management and protocol deviations could be attributed to poor training and knowledge, deficiencies in monitoring and the lack of clinical governance [37]. They reported consistently poor information sharing by the staff; and the importance of returning for TB results was only discussed in a third of interactions. In this study, health promotion talks at facilities addressed generic information on TB symptoms and infection control measures, encompassing how treatment completion ensured better health and prevented resistant disease, cough hygiene practices and early TB symptom identification and side effect diagnosis and management. There was no standardised guideline from which this information was drawn, and the message varied across clinicians and facilities. Consequently, there was no objective measure of whether the patients had been adequately informed about the disease or its management. The overall findings from the Western Cape study suggested low effort in communicating invaluable information regarding untreated TB and the need for treatment and acknowledged that the poor adherence to the protocols were possibly resulting in the disappointing TB outcomes [37].

Secondly, the reception of information by patients warrants critical appraisal. In the best-case scenario, knowledge leads to understanding of taking informed action, but this does not necessarily lead to the action [38] for a multitude of reasons. Reasons ranging from access, loss of income, stigma, side effects and pill burden have long been implicated [39, 40]. The health literacy of patients has relevance in this context where patients have interrupted treatment based on subjective improvement in health. Studies from Malawi and Zambia showed that patients advised to refrain from beer, smoking and frequent sex during their TB treatment, without proper explanation of these restrictions, were likely to reconsider their presentation for treatment [38, 41]. Patient’s preference for a treatment facility was rarely discussed during the consultation and negotiation of care was not facilitated. Thus, despite DOT supporters being on duty at some facilities, the clinicians found that tracing of LTFU patients was a futile exercise given the false addresses provided by the patients. As a result, facilities were made reliant on LTFU patients self-presenting, after not been located by treatment supporters.

Finally, the insufficiencies of the patient-clinician relationship are brought into question. Patient counselling remains a critical element in reducing LTFU [38] reducing LTFU pointing to integrated behavioural counselling and TB care promoting adherence to treatment protocols [41, 42]. Clinicians require knowledge of TB disease and its management and excellent communications skills to ensure quality patient education and communication [43, 44]. User-friendly and culturally appropriate resources presented in local languages are a potential approach. The use of simple, written information, enhanced by visuals cues like pictograms, have been shown to enhance comprehension and recall of information [45–47]. The use of a pictorial-based TB booklet in primary health care clinics in the Eastern Cape, South Africa was perceived as an invaluable tool in patient education [48]. Illustrated information has been shown to strengthen patient communication by the provision of accurate information that promotes adherence to guidelines and minimises guesswork [49]. Whether it is with the use of new resources or in day-to day care, clinicians need adequate training in communication, particularly when the interaction is linked to treatment adherence [50]. Emerging mHealth technologies could serve to bridge this patient-clinician communication gap and reduce LTFU [51, 52] through text messaging for example [24, 53]. However, this strategy is reliant on the access to mobile technology and the availability of current contact details which have proved dubious in this study. Arulchelva et al. conducted similar work in India and encouraged the use of mass media, such as playing health related videos on mobile phones, in addition for personal communication [54].

Most TB patients designated LTFU cited the underlying inability to negotiate their treatment plans as the reason for the interruption in care. It appears that the needs of the clinician during the consultation, described as efficiently relaying information on TB treatment and adherence, were met. However, whether this information appropriately addressed the patients concerns and expectations appears only in the context of case-finding in the literature [55–60] and not on treatment outcomes in South Africa and globally. Similar findings from a previous study demonstrated that despite standardised guidelines effectively contributing to health coverage; ignoring the subjective biopsychosocial aspects of care particularly in rigid health systems resulted in ‘dehumanising’ the care process [25].

Adopting the patient centered approach is acknowledged to be directly beneficial to patients as it allows the clinician to reach an understanding of the patient’s expectations, feelings and social context of the illness [61]. The clinician that assumes the role of expert but remains an equal in the relationship is likely to achieve better treatment outcomes. Discussion of management plans with the patient will allow for their preferences to be considered, discussed and incorporated; thereby adapting care to suit the individualised needs of the patient. More widely, the patient centred approach focusses on empowering TB patients and their communities, social support programmes and enhanced communication and partnership between community and health sectors [62].

### Strength and limitations

An important strength of this study is the study being based in a high-density population, with a significant TB disease burden. Further, the opinions of both the patients and clinicians were sought allowing for the exploration of TB treatment adherence from differing perspectives. This study explored the reasons for discontinuing TB treatment by approaching those who had stopped TB treatment. However, the absence of the views of those who had successfully completed treatment, for the purposes of meaningful comparison, may be considered a limitation of this study. We were unable to access all the patients that were LTFU as they had moved away, provided incorrect contact details or the data was missing. Their inclusion may have yielded additional insights. Potentially, some of the responses obtained may have been limited given the sensitive nature of TB and its association with HIV, which also carries a significant burden of stigma and discrimination. The study was limited to a single district in the Free State Province, thus the findings cannot be extrapolated to other settings.

## Conclusions

This study reiterates the need for exploring the critical socio-cultural interface between clinician and patients in order to achieve improved TB outcomes rather than the current dependence on biomedical interventions. Enhancing communication and developing innovative strategies to convey TB messaging to patients and the community should remain a critical focus of the clinician. Further research is warranted in developing early warning systems in identifying those at risk of treatment interruption and in developing and testing communication tools that actively promote treatment adherence. Lastly, it is vitally important to acknowledge the crucial role nurse clinicians play in TB management and control, thus ensuring they are suitably trained cannot be over-emphasized. TB remains a social disease, the successful management of which hinges on patient-centred care.

## Supplementary information

**Additional file 1: Annexure 1.** Patient Interview Guide. **Annexure 2.** Clinician Interview Guide. **Annexure 3.** Characteristics of Participants. **Annexure 4.** Patient Recruitment Strategy.

## Data Availability

The datasets used and analysed during the current study are available from the corresponding author on reasonable request.

## Abbreviations

AIDS: Acquired immunodeficiency syndrome
DOTS: Directly observed treatment, short course
GCP: Good Clinical Practice
HIV: Human immunodeficiency virus
IDI: In-depth interviews
LTFU: Loss to follow up
PHC: Primary health care
SD: Standard deviations
TB: Tuberculosis
WHO: World Health Organization
