# Supplementary material for: ‘They are inconveniencing us’ - exploring how gaps in patient education and patient centred approaches interfere with TB treatment adherence: perspectives from patients and clinicians in the Free State Province, South Africa
Source: BMC Public Health. 2020 Apr 6;20:454. doi: 10.1186/s12889-020-08562-3 (PMC7137430; doi:10.1186/s12889-020-08562-3)
Supplement: Supplementary file 1 — Additional file 1: Annexure 1. Patient Interview Guide. Annexure 2. Clinician Interview Guide. Annexure 3. Characteristics of Participants. Annexure 4. Patient Recruitment Strategy. [file 12889_2020_8562_MOESM1_ESM.docx]

**Annexure 1: Patient Interview Guide**

**STUDY NUMBER**: ---

**Please read this out to the Participant before starting interview**

- Thank you for agreeing to take part in our research.
- We are interested in knowing about people’s health. Some of these questions are sensitive, but it is very important that we know the truth about what is going on in this community. There are no right or wrong answers.
- You do not have to answer any questions if you don’t want to. Just say pass and I will move to the next question.
- Your answers to these questions may help us make services better.
- All of your answers will be kept confidential. Confidential means we will not tell your answers to anyone outside the research group.
- Your answers in the interview will only be identified by an assigned study number and not your name.
- First we are going to ask a few questions about you. Then we will move to questions about your views.

**Socio-demographic Questions**

| **Variable** | **Options** |
| --- | --- |
| 1. Gender | Male / female |
| 1. Age | In years (continuous) |
| 1. Education | None/primary/secondary/ tertiary |
| 1. Marital status | Single/married/divorced/widowed |
| 1. Source of income | No income/self-employed (state)/salaried or wages/social grants |
| 1. Household income | ≤ R1000, >R1000, >R5000 |
| 1. Health services | Public/private/public and GP |
| 1. Distance from clinic | In metres or km (continuous) |
| 1. Water supply | Piped / communal tap / no water source |
| 1. Electricity | Electricity / paraffin /coal, wood |
| 1. Sanitation | Toilet in house / communal toilet / pit latrine / none |
| 1. Has a contact number | Yes / no |
| 1. Medical conditions | DEATH mental other |
| 1. Alcohol history | Yes / no, if yes – years, frequency, amount |
| 1. Smoking history | Yes / no, if yes – years, frequency, amount |

**Now we will move on to questions about your views.**

**ACCESS TO HEALTHCARE**

- How often do you access clinic services?
- Please tell us about the type of clinic services that you access?
- In terms of TB services, please describe your experience of attending the clinic?
- Can you describe to us, how you usually get to the clinic?
- Describe any challenges accessing clinic services?
- How did the staff treat you when you were accessing TB services and how did this make you feel?
- Please tell us about any positive experiences that you had when attending the clinic
- If you had to tell us how to improve access to healthcare specifically to TB treatment, what would you recommend?

**PERCEIVED QUALITY OF CARE**

- Please describe to us your understanding of your TB diagnosis and treatment requirements
- When you visited the clinic, please tell us who and how they explained your TB condition to you?
- Describe to us if the information provided to you met your expectations when compared to the actual TB services that you received
- What were some of the things that you disliked when being treated for TB?
- How could we change service delivery to ensure that your experience of TB treatment is positive?

**KNOWLEDGE OF TB**

- Have you heard about a disease called TB (tuberculosis)? Can you explain to us the type of information on TB that you heard about?
- Do you know how tuberculosis can be spread? If yes, tell me more.
- How did you find out that you had TB?
- Can you tell us about your understanding of TB treatment? For example, how long is the treatment recommended in order to cure the disease?
- Explain to us your understanding of the side effects of TB.
- Have you experienced any of these side effects and what have you done to overcome this?
- Please tell us about any other illness that you have been diagnosed with and all the types of medication that you are taking for these (Probe on when medication is taken)

**TREATMENT SUPPORT**

- Can you talk about TB in your house? If yes or no, what are some of the reasons?
- Who do you usually confide in regarding your health?
- What were the reasons for disclosing or keeping your TB diagnosis a secret?
- How did your family and community treat you when they found out about your TB diagnosis?
- Can you tell us who may have treated you differently when they found out about your TB diagnosis and what did they do?
- How did your behaviour change when you found out that you had TB?
- What type of methods are in place to remind you to take your medication for TB?
- How do you feel about taking TB medication daily?
- Can you describe to us how you managed with taking medication daily?
- What were the factors that stopped you from taking medication daily?
- What were the factors that helped you to take your medication daily?

**UNDERSTANDING OF LOST TO FOLLOW-UP**

- Can you describe to us the reasons why you remained on treatment or stopped taking your medication?
- Please tell us what were the good things about being on treatment or taking your medication
- Please tell us what were the difficult things about being on treatment or taking your medication
- Can you tell us what effect the TB medication had on your body?
- What are some of the factors that influenced you to stop taking medication?
- Can you describe to us some of reasons for missing visits?
- Can you describe any recommendations on how we could encourage people to stay on TB treatment?

**Annexure 2: Clinician Interview Guide**

**STUDY NUMBER**: ---

**Please read this out to the Participant before starting interview**

- Thank you for agreeing to take part in our research.
- We are interested in knowing about people’s health. Some of these questions are sensitive, but it is very important that we know the truth about what is going on in this community. There are no right or wrong answers.
- You do not have to answer any questions if you don’t want to. Just say pass and I will move to the next question.
- Your answers to these questions may help us make services better.
- All of your answers will be kept confidential. Confidential means we will not tell your answers to anyone outside the research group.
- Your answers in the interview will only be identified by an assigned study number and not your name.
- First we are going to ask a few questions about you. Then we will move to questions about your views.

**INTRODUCTORY QUESTIONS**

First a few questions about you and your work situation.

1. How old are you?
2. What is your home language?
3. What is your job title?
4. How long have you been working as a clinician?
5. How long have you been working at this facility?

Now we will move to questions about your views.

**Standard of care**

1. What services are offered at your facility to assist patients with taking TB treatment?
2. Please describe your training on managing TB. Has your experience impacted the manner in which you manage TB patients?
3. List the guidelines you use to diagnose TB at your facility.
4. What problems have you experienced at your facility when offering of TB services?
5. Can you describe the management/maintenance of TB records at your facility?

**Patients Understanding of disease**

1. What is your perception of your patient’s knowledge regarding TB?
2. Please describe your positive and negative experiences with patients taking TB treatment.
3. How do you think patient’s knowledge of TB influences them to continue treatment or stop treatment?
4. From your experience, what are the factors that contribute to patients either continuing with or stopping treatment?

**Facilitators and barriers to TB treatment**

1. What challenges do you think patients face in completing TB treatment?

2. Why do you think some patients fail to complete TB treatment?

3. Why are some patients successful in completing the full duration of treatment?

4. What do you think can be done at your facility to improve the completion rate of TB treatment?

**ANY OTHER COMMENTS**

**Annexure 3: Characteristics of Participants**

**Table 1. Codes and characteristics of patients (N = 7)**

| **Code #** | **Clinic** | **Gender** | | **Age** | **Education** | **Marital Status** | **Comorbidities** | **Distance to Facility (km)** |
| --- | --- | --- | --- | --- | --- | --- | --- | --- |
| 1 | MUCPP CHC | F | 80-89 | | None | Separated | Epilepsy | 0-10 |
| 2 | MUCPP CHC | M | 20-29 | | None | Single | HIV on ART | - |
| 3 | MUCPP CHC | M | 40-49 | | None | Single | Nil | 11-20 |
| 4 | Pelonomi Polyclinic | M | 40-49 | | Secondary | Single | Nil | 0-10 |
| 5 | Pelonomi Polyclinic | M | 20-29 | | Secondary | Single | HIV on ART | 11-20 |
| 6 | Pelonomi Polyclinic | M | 20-29 | | Secondary | Married | Nil | 0-10 |
| 7 | Pule Sefatsa PHC | M | 20-29 | | Secondary | In a relationship | Nil | 0-10 |

**Table 2: Codes and characteristics of clinicians (N = 15)**

| **Code #** | **Facility** | **Gender** | **Age** | **Home Language** | **Job Title** | **Duration of Employment (years)** |
| --- | --- | --- | --- | --- | --- | --- |
| 8 | MUCPP CHC | F | 40-49 | Sotho | Doctor | 11-20 |
| 9 | MUCPP CHC | F | 40-49 | Xhosa | Assistant Nurse | 0-10 |
| 10 | MUCPP CHC | F | 40-49 | Sesotho | Enrolled Nurse | 0-10 |
| 11 | MUCPP CHC | F | 40-49 | Sesotho | Professional Nurse | 11-20 |
| 12 | MUCPP CHC | F | 40-49 | Sesotho | Enrolled Nurse | 0-10 |
| 13 | Pelonomi Polyclinic | M | 20-29 | Sesotho | Professional Nurse | 0-10 |
| 14 | Pelonomi Polyclinic | F | 50-59 | Sesotho | Professional Nurse | 21-30 |
| 15 | Pelonomi Polyclinic | F | 50-59 | Sesotho /Xhosa | Operational Manager | 21-30 |
| 16 | Pelonomi Polyclinic | F | 40-49 | Xhosa | Professional Nurse | 21-30 |
| 17 | Pelonomi Polyclinic | M | 40-49 | Setswana | Doctor | 11-20 |
| 18 | Pule Sefatsa PHC | F | 50-59 | Sesotho | Professional Nurse | 21-30 |
| 19 | Pule Sefatsa PHC | F | 40-49 | Xhosa | Professional Nurse | 11-20 |
| 20 | Pule Sefatsa PHC | F | 20-29 | Sesotho | Professional Nurse | 0-10 |
| 21 | Pule Sefatsa PHC | F | 40-49 | Xhosa | Professional Nurse | 0-10 |
| 22 | Pule Sefatsa PHC | F | 50-59 | Sesotho | Professional Nurse | 31-40 |

**Annexure 4: Patient Recruitment Strategy**

Recruitment Strategy

Patients with TB Outcome of LTFU identified on ETR.net & EDR.web for MUCPP CHC

Patients with TB Outcomes of LTFU identified on ETR.net & EDR.web for Pule Sefatsa PHC

Patients with TB Outcome of LTFU identified on ETR.net & EDR.web for Pelonomi Polyclinic

163 LTFU identified

103 LTFU identified

2 LTFU identified

2 Patient Files retrieved

20 Patient TB Files retrieved

49 Patient TB files retrieved

1= Deceased

1= Agreed to be interviewed

17=could not be contacted

3=Agreed to be interviewed

44=could not be contacted

3=Agreed to be interviewed

1=Deceased

1=Moved

7 Interviews Conducted
